# Perceived barriers and facilitators of implementing a sustained smartphone-based telemonitoring program for pregnant women at high-risk for pre-eclampsia in the public and private sectors in Pakistan

**DOI:** 10.1177/20552076241292682

**Published:** 2024-12-10

**Authors:** Anam Shahil-Feroz, Andaz Riaz, Haleema Yasmin, Sarah Saleem, Zulfiqar Bhutta, Emily Seto

**Affiliations:** 1 Community Health Sciences Department, 9615 Aga Khan University, Karachi, Pakistan; 2 Dalla Lana School of Public Health, 7938 The University of Toronto, Institute of Health Policy Management and Evaluation, Toronto, ON, Canada; 3 Department of Obstetrics and Gynecology, 443365 Jinnah Postgraduate Medical Center, Karachi, Pakistan; 4 Centre for Global Child Health, SickKids, Toronto, ON, Canada; 5 Center of Excellence in Women and Child Health, 9615 Aga Khan University, Karachi, Pakistan; 6 Dalla Lana School of Public Health, 7938 The University of Toronto, Toronto, ON, Canada; 7 Centre for Digital Therapeutics, University Health Network, Toronto, ON, Canada

**Keywords:** Telemonitoring, Pakistan, self-monitoring, reproductive health, qualitative studies, pregnancy medicine

## Abstract

**Background:**

In Pakistan, a smartphone-based telemonitoring (TM) program (Raabta) has been designed to support pregnant women with high risk for preeclampsia (HRPE) in Pakistan. However, implementing TM interventions is often challenging, particularly in low-resource settings, given the complexity of healthcare environments and variations in public and private health sectors. This study explores the potential barriers and facilitators for a sustained implementation of the Raabta program in public and private sector hospitals in Pakistan.

**Methods:**

Using a qualitative description design, 57 semi-structured interviews with a diverse group of participants including patients from the public (*n = *15) and private sector hospitals (*n = *17), obstetricians from the public (*n = *5) and private sector hospitals (*n = *7), decision-makers (*n = *7) and telehealth experts (*n = *6). Participants were recruited using purposive and snowball sampling techniques. Interview transcripts were deductively analyzed using the Consolidated Framework for Implementation Research (CFIR) domains.

**Results:**

Based on the CFIR domains, the findings included: (1) Raabta being perceived as user-friendly even for patients with low digital and language literacy; (2) Outer settings: Limited health and digital literacy, poor language proficiency, and cultural norms can influence the willingness and ability of public sector patients to use the Raabta; (3) Inner settings: The private health sector is well-equipped for the Raabta implementation, while the public health sector faces challenges related to physical space, limited human and financial resources, and physician resistance; (4) Individual characteristics: Majority participants demonstrated positive attitudes toward the Raabta program and expressed confidence in using it (5) Process: Recommendations included adopting a nurse-led model for the private sector, leveraging paramedics for monitoring the Raabta dashboard, integrating Raabta with existing digital platforms, and establishing an advisory committee for program sustainability.

**Conclusion:**

Raabta implementation may be more feasible in the private sector due to patient demographics, health and digital literacy, cultural norms, financial resources, physician readiness, and hospital infrastructure.

## Introduction

In Pakistan, preeclampsia/eclampsia (PE/E) is responsible for approximately one-third (34%) of maternal deaths in tertiary-level healthcare facilities.^1^ The high maternal morbidity and mortality from PE/E stems from a lack of early risk detection, diagnosis, and treatment of high-risk pregnant women.^2–6^ Telemonitoring (TM) has emerged as a potential tool to support pregnant women at high risk for preeclampsia (HRPE).^7–11^ TM for PE involves pregnant women monitoring their blood pressures at home with the help of devices (e.g., mobile phone apps and blood pressure machines) for real-time monitoring by providers.^9^ Previous studies have recommended TM for high-risk pregnant women because of its benefits such as better blood pressure control, early risk identification and treatment, fewer hospital visits, and cost-savings.^12–15^ However, most TM interventions for supporting high-risk pregnant women have been implemented in high-income countries, such as the UK, the USA, and Belgium,^8–10,14–18^ with a paucity of evidence on its use and effectiveness in LMICs.^19^

Considering the potential of TM, we have developed a smartphone-based TM program called “Raabta.” The name “Raabta” derives from the Urdu language, meaning “connection.” The Raabta technology is designed to empower pregnant women at HRPE to self-monitor their blood pressure and symptoms at home. This allows them to receive automated self-care alerts and enables real-time monitoring by clinicians between antenatal visits. The development of the Raabta program has been informed by our foundational work, including a scoping review,^19^ two needs assessment studies,^20,21^ and a usability study of the Raabta program.^22^ The Raabta program consists of a patient-facing technology, which includes a smartphone app and Bluetooth-enabled blood pressure device for daily monitoring and alerts, and a clinician-facing web-based dashboard for clinical decision-making using patient data collected from the Raabta app.^23^

A consistent finding in clinical and health services research is the failure to translate evidence into practice.^24^ The implementation of TM programs in healthcare environments is complex, influenced by dynamic interactions between the intervention, stakeholders, and the overall context of the implementation setting.^25,26^ TM implementation is particularly challenging in low-resource settings due to differences in how public and private sectors are organized and function. The healthcare landscape in Pakistan is marked by a significant reliance on the private sector, which accounts for 85% of total health expenditure and 81% of out-of-pocket (OOP) costs, while the public sector primarily serves low-income and underserved populations.^27,28^ The public sector faces considerable challenges, such as inadequate funding (less than 1% of GDP), infrastructure deficits, and workforce shortages, which hinder its ability to provide comprehensive care.^29^ In contrast, the private sector, perceived to offer higher quality services, is financially burdensome for low-income individuals due to high OOP expenses and limited insurance coverage.^30^ The private healthcare sector remains the preferred choice for 80% of MNCH-related services in Pakistan.^30^ Several factors contribute to the slow progress of MNCH in the country, including social determinants such as poverty, high illiteracy rates, lack of women's and girls’ empowerment, and high fertility rates.

To ensure the successful implementation of the Raabta program, it is recommended that the readiness for implementation be assessed and the barriers and facilitators be identified in advance.^31^ This study aims to examine the implementation of the Raabta program in both public and private sectors to explore factors such as patient demographics, funding models, resources, organizational structures, regulatory considerations, and implementation climate including institutional and provider needs. The study aims to explore challenges and facilitators to inform context-specific strategies for successful implementation in diverse healthcare settings.

## Methods

This study is part of a larger research project focused on developing and implementing the Raabta program for pregnant women at HRPE in Karachi, Pakistan, as a proof of concept.^23^ Prior to the implementation of the Raabta program for a feasibility study,^23^ the aim was to gain a comprehensive understanding of the potential barriers and facilitators associated with the sustained implementation of the Raabta program in public and private sector hospitals in Pakistan.

### Study intervention: smart-phone telemonitoring program named “Raabta”

The study intervention includes a Raabta smartphone application (app), a Bluetooth-enabled blood pressure device validated for use in pregnancy, and a clinician web-based Raabta dashboard. The patient-facing Raabta app enables pregnant women to take daily blood pressure readings at home with a provided Bluetooth-enabled home blood pressure monitor and to answer one yes/no symptom question. The app provides automated alerts (i.e., self-care messages) to women based on the entered data. The clinician-facing technology includes the web-based Raabta dashboard, allowing clinicians to monitor alerts intermittently and support clinical decision-making. The patient and clinician alerts are generated though a rules-based algorithm, which was developed using NICE guidelines^32^ and through consultations with expert obstetricians in Pakistan.

### CFIR application: prior to the implementation of the Raabta program

Implementation research without a theoretical basis limits the understanding of implementation conditions across different contexts.^11,33–36^ Our study used the Consolidated Framework for Implementation Research (CFIR), an established implementation framework, to enable a richer understanding of the barriers and facilitators associated with the potential implementation of the Raabta program in public and private sector hospitals in Pakistan. The framework can be applied to any implementation phase (i.e., pre-, during, or post-implementation). Our study used the CFIR prior to the implementation of the Raabta program to enable a structured investigation of barriers and facilitators for the potential implementation of the Raabta program. The CFIR domains for the current study included: (1) knowledge and beliefs of patients, obstetricians, decision-makers, maternal health and telehealth researchers/experts (Characteristics of Individuals); (2) characteristics of the Raabta program (Intervention Characteristics); (3) structural characteristics and implementation climate of the public and private sector (Inner Setting); (4) needs and resources of the pregnant women (Outer Setting); and (5) the preparation for the implementation of the Raabta program (Process).^31^ Our study selected CFIR constructs that were most relevant to guide the assessment of potential barriers and facilitators for the sustained implementation of the Raabta program at public and private sector hospitals in Pakistan. For instance, CFIR construct “Execution” (Implementation Process) is excluded from this analysis since the Raabta program has not yet been implemented. This construct will be addressed in future evaluations once the program is actively rolled out.

### Study design, setting and participants

We employed a qualitative description study design to explore the potential barriers and facilitators associated with the sustained implementation of the Raabta program in public and private sector hospitals in Pakistan. The study was conducted in Pakistan from October to December 2023. The study involved a diverse group of participants including patients from the public and private sector hospitals, obstetricians from the public, and private sector hospitals, decision-makers and telehealth experts. Purposive and snowball sampling techniques were used to recruit obstetricians, decision-makers, and telehealth experts from various public and private sector hospitals and organizations to ensure diversity in gender representation, years of experience, and inclusion of major institutions from both sectors. Decision-makers were also identified through health ministries at both the national and provincial levels in Pakistan. Pregnant patients at HRPE were purposively sampled from a private sector hospital (Ob-Gyn outpatient department at AKUH) and a public sector hospital (Ob-Gyn outpatient department at JPMC) to ensure a diverse sample in terms of maternal age, gestational age, education status, occupation, gravidity, and parity.

Ethical approvals were obtained from the University of Toronto Research Ethics Board (45007), AKU (2023-9010-27332) and the National Bioethics Committee (981) in Pakistan. Written informed consent was obtained from all participants prior to interviews.

### Participant recruitment and eligibility criteria

The study recruited eligible obstetricians, decision-makers, telehealth experts, as well as private sector patients to understand the barriers and facilitators associated with the potential implementation of the Raabta program at public and private hospitals in Pakistan. Table 1 defines pregnant women at HRPE as per the NICE guidelines. Our sampling approach included participants from both public and private sectors, ensuring a balanced representation and capturing determinants of implementation specific to each sector. Obstetricians from public and private sector hospitals, telehealth experts, and decision-makers including hospital administrators and officials at the provincial and national ministry were initially identified and recruited with the assistance of the local study team in Pakistan. Additional participants were identified through snowball sampling. These participants were invited to participate in the qualitative study via email and were requested to sign informed consent forms before the interview began.

**Table 1.** Definition of pregnant women at high risk for pre-eclampsia as per the national institute for clinical excellence guidelines.^27^

Definition NICE guidelines define pregnant women at HRPE as those who have 1 high-risk factor or >1 moderate risk factor for PE. *High-risk factors:* Hypertensive disease in a previous pregnancy Chronic kidney disease Autoimmune diseases, such as systemic lupus erythematosus or antiphospholipid syndrome Type 1 or type 2 diabetes Chronic hypertension *Moderate risk factors:* First pregnancy Aged ≥40 years Pregnancy interval of >10 years BMI of ≥35 kg/m^2^ at the first visit Family history of PE Multifetal pregnancy

The recruitment of patient participants from the private sector was supported by nurses at the AKUH. Nurses first identified eligible pregnant women from the outpatient setting of the Ob-Gyn department at AKUH. Once a potential patient participant was identified, the nurses contacted the on-site facilitator, who then reached out to the identified participant to explain the study’s purpose and procedures and to inquire about their willingness to participate. If the patient participant was unable to read the consent form, the on-site facilitator explained the consent form verbally in their local language. Pregnant women at HRPE who were unable to write their names were asked to provide a thumbprint to mark their consent to participate. Patients from public sector hospitals were previously interviewed during needs assessment,^20^ and their insights on the barriers and facilitators to implementation of Raabta were incorporated into this current study.

Table 2 provides a list of the eligibility criteria for the patient participants and the key informants.

**Table 2.** Eligibility criteria for pregnant patients, obstetricians, decision-makers, and telehealth experts.

Pregnant Patients *Inclusion criteria:* Pregnant women at HRPE who were visiting the AKU Ob-Gyn outpatient department to seek antenatal care Pregnant women at HRPE who met the NICE guidelines definition of HRPE Pregnant women who were diagnosed as HRPE for at least 3 weeks and have had time to reflect on the disease condition, associated difficulties, and need for TM Pregnant women with the ability to speak English, or Urdu language *Exclusion criteria:* Pregnant women at HRPE who were admitted to the inpatient wards and emergency care for treatment purpose Obstetricians, Decision-Makers, and Telehealth Experts *Inclusion criteria:* Obstetricians, decision-makers, and telehealth experts who were directly or indirectly involved in the care of pregnant women at HRPE Ability to speak Urdu or English language

### Data collection

After obtaining consent from the participants, the on-site facilitator and patient participants moved to a quiet area within the AKUH outpatient department for in-depth interviews. The interview was conducted immediately after recruitment, while the participants were waiting to see their doctor for a routine clinic visit. The interview was conducted via Zoom (Zoom Video Communications, Inc) in the Urdu language by a primary investigator (ASF), located in Toronto, Canada. The primary investigator had experience conducting qualitative research in Pakistan and was familiar with the culture, population, and language. An on-site facilitator assisted with obtaining informed consent, setting up the Zoom link, and accompanying the patient. The interviews with obstetricians, decision-makers, and telehealth experts were conducted in English and via Zoom. Prior to the interviews, all study participants received a demonstration of the Raabta app through screen sharing via Zoom. This demonstration provided an overview of how the app functions, including the tasks involved and how women will receive self-care alerts. Following the demonstration, participants had the opportunity to ask questions and were offered a repeat demonstration upon request. This approach was designed to help them understand the Raabta app better and to provide insights into the barriers and facilitators related to its potential implementation and sustainability. The interviews lasted between 40 and 60 min. The interviews were audio-recorded and later transcribed (and patient interviews were translated to English) by a professional transcriptionist for analysis. Data collection and analysis occurred simultaneously to determine the point of the data saturation.^37,38^

The interviews followed a semi-structured guide developed using the CFIR (source: https://cfirguide. org/).^31^ The interview guide questions were tailored to accommodate the unique roles or tasks of different groups of participants (see Appendix).

### Data analysis

The interview transcriptions were uploaded into the NVivo software for analysis using the framework approach.^39^ The framework analysis followed the five steps: (1) familiarization, (2) identifying a thematic framework, (3) indexing, (4) charting, and (5) mapping/interpretation. The analysis involved an ongoing iterative process where the primary investigator (ASF) conducted several reviews of the transcripts to become familiar (Step 1) with the data and recognize preliminary reflexive and interactive themes. Data analysis began immediately after the first interview was completed and continued concurrently with data collection to establish the data saturation point. Although our research used CFIR as the a priori framework (Step 2), the researchers were cognizant of identifying additional codes falling outside of CFIR. Codes relevant to CFIR constructs across the five domains were indexed (Step 3) to the entire transcripts using NVivo. Portions of the transcripts were charted into themes using NVivo (Step 4). The themes were organized by CFIR domains and constructs and then restructured to better reflect descriptions from study participants. All these steps were carried out simultaneously by two independent researchers (ASF and AR). Initially, both researchers met to establish a consensus on the codes and themes. Subsequently, the entire research team reviewed the codes, categories, and themes multiple times to check for possible biases and enhance the credibility of the interpretation (Step 5) of the interviews.

## Results

A total of 57 semi-structured interviews were conducted to explore the barriers and facilitators for the implementation of the Raabta program in both public and private hospitals in Pakistan. Interviews were conducted with a diverse group of participants including patients from private sector hospitals (*n = *17), patients from the public sector (*n = *15), obstetricians from the public (*n = *5) and private sector hospitals (*n = *7), decision-makers (*n = *7) and telehealth experts (*n = *6). A total of 77 pregnant patients at HRPE were approached by the study team, of which 20 patients refused to participate. Reasons for nonparticipation included patients not willing to wait after the clinic visit, being unable to get permission from caregivers, having children waiting at home, participants feeling unwell, and a general lack of willingness to participate in the study.

The demographic information for all the obstetricians, decision-makers, and telehealth experts as well as patient participants is illustrated in Tables 3 and 4, respectively.

**Table 3.** Characteristics of obstetricians, decision-makers, and telehealth experts (*N* = 25).

Characteristics and Category		Values
Gender, *n* (%)		
	Female	16 (64)
	Male	9 (36)
Role, *n* (%)		
	Obstetricians from Public Sector (OB-GYN)	5 (20)
	Obstetricians from Private Sector (OB-GYN)	7 (28)
	Decision-makers	7 (28)
	Telehealth experts	6 (24)
Experience		
	Years, mean (SD)	24.16 (8.59)
	Years, median (range)	24 (8–40)

**Table 4.** Characteristics of patient participants from private (*N* = 17) and public sector (*N* = 15).

Characteristics and Category		Private Sector	Public Sector
Gender, *n* (%)			
	Female	17 (100)	15 (100)
Age (years)			
	Years, mean (SD)	27.33 (4.38)	28.26 (5.28)
	Years, median (range)	28 (20–35)	28 (20–38)
Highest educational level, *n* (%)			
	No education	0	3 (20)
	Less than high school	6 (35.29)	3 (20)
	High school	2 (11.76)	8 (53)
	University	9 (52.94)	1 (7)
Occupation, *n* (%)			
	Housewife	12 (70.58)	11 (73)
	Professional	5 (29.41)	4 (27)
History of PE, *n* (%)			
	Yes	2 (11.76)	9 (60)
	No	15 (88.23)	6 (40)
Pregnancy (weeks)			
	Weeks, mean (SD)	28.28 (5.19)	31.67 (6.06)
	Weeks, median (range)	29 (15–34)	33 (12–37)
Access to a personal home blood pressure machine, n (%)			
	Yes	15 (88.23)	2 (13)
	No	2 (11.76)	13 (87)
Gravida,^a^ *n* (%)			
	Primigravida	7 (41.17)	4 (27)
	Multigravida	10 (58.82)	11 (73)
Parity,^b^ *n* (%)			
	Nullipara	8 (47.05)	4 (27)
	Primipara	9 (52.94)	9 (60)
	Grandpara	0	2 (13)
Personal or shared access to a smart phone, *n* (%)			
	Personal access	17 (100)	9 (60)
	Shared access	0	6 (40)
Access to the internet, *n* (%)			
	Yes	17 (100)	8 (53)
	No	0	7 (47)

The barriers and facilitators associated with the potential and sustained implementation of the Raabta program are summarized in Table 5. A total of 14 relevant CFIR constructs were identified across five domains to explain these factors: two constructs in each of the domains of individual characteristics, inner setting, outer setting, and implementation process, and six constructs in the domain of intervention characteristics. Given that the study was structured using the CFIR framework, the findings are categorized under the five key domains of CFIR, which serve as the main themes for the analysis.

**Table 5.** Potential barriers and facilitators for raabta implementation.

Domains	Barriers	Facilitators
Domain 1: Characteristics of Individuals	Potential Implications for Providers for Not Responding to Alerts (Knowledge and Beliefs) Perceived challenges for Sustaining Raabta Program in the Public and Private Sector (Knowledge and Beliefs)	Perceived Reduced Hospital Burden and Enhanced Patient Experience (Knowledge and Beliefs) Perceived Self-efficacy Among Obstetricians in the Ability to Use Raabta Program (Self-efficacy)
Domain 2: Intervention Characteristics	Language Inclusivity (Adaptability) Need Additional Diagnostic Parameters (Adaptability) Technical Challenges Associated with Raabta Program Use (Complexity) Concerns for Patient Safety (Complexity) Additional Costs Associated with Continuous Monitoring (Costs)	Raabta Program Designed using Evidenced-based Guidelines (Evidence strength and quality) Convenient, Systematic, and Easy-to-Use Program than the Current Practice (Relative Advantage) Positive Feedback on Raabta Features (Design Quality & Packaging) Pilot Trial of Raabta Program (Trialability)
Domain 3: Inner Setting	Infrastructure and Resource Constraints in Public Sector (Structural Characteristics) Resistance to Change Among Obstetricians in Public Sector (Implementation Climate) Government Hesitancy to Implement Raabta in the Public Sector (Implementation Climate)	Leveraging Established Infrastructure of the Private Sector (Structural Characteristics) Endorsement from Department Leads (Implementation Climate) Dedicated Resource for Monitoring Clinician Dashboard (Implementation Climate) Incentives for Obstetricians (Implementation Climate)
Domain 4: Outer Setting	Perceived lack of compliance with the Raabta program among public sector patients (Patient Needs & Resources)	Supportive Policy and Funding Landscape (External Policy & Incentives)
Domain 5: Implementation Process		Positioning Raabta within Existing MNCH Service Packages and Digital Platforms (Planning) Raabta as a Prescription by Physicians (Planning) Engaging Key Opinion Leaders for Implementation Success (Engaging) Building an Advisory Committee for Raabta Program Sustainability (Engaging)

### Domain 1: characteristics of individuals

All participants, including patients, obstetricians, telehealth experts, and decision-makers, uniformly conveyed a positive attitude toward the Raabta program and recognized several potential benefits compared with the current practice. For instance, participants perceived the Raabta program as valuable for reducing patient travel distances and easing the strain on emergency rooms, offering patients the opportunity to return home safely instead of enduring extended hospital stays. In general, the Raabta program was perceived as highly beneficial by all participants for improving the quality of blood pressure measurement and remote management of high-risk pregnancies to improve maternal health outcomes.

If a woman comes to the usual clinic and her blood pressure is obviously elevated after waiting for so long, especially in hot and humid weather, reaching 140/90, usually we ask them to go to the emergency and stay there as a day care option… through Raabta, we can avoid such situations, and I believe it will decrease the burden on the emergency rooms in public hospitals. (Public Sector Obstetrician 03)

A subset of obstetricians, both in the public and private sectors, highlighted possible consequences should providers fail to respond promptly to alerts. This includes the potential legal accountability for providers in cases where delayed responses to alerts lead to harm or adverse outcomes.

…we deal with a variety of patients, … we have competing responsibilities in the hospital, academics, and clinics, we might not have the dedication or time to go through that screen and take on such a huge responsibility. The patient may claim that they sent us a message, and if we didn't see it, then we are responsible for everything. Speaking for myself, I wouldn't want to take this responsibility because then I would be accountable if the patient faces adverse outcomes.

(Private Sector Obstetrician 05)

When asked about the sustained implementation of the Raabta program in the public and private sector hospitals, it was mentioned that pregnant women and family caregivers in the private sector will be more likely to adopt and use the Raabta program for remote monitoring due to fewer financial and cultural barriers. Whereas, the public sector will face difficulties in using the Raabta program due to socioeconomic, cultural, financial, and accessibility barriers, limiting the reach and sustainability of the program.

I have my doubts regarding this because women are not allowed to use mobile phones very independently. … They are not given access to phones as much as the male members of the family, I don't think it would be very feasible for the long run. (Public Sector Obstetrician 02)

Obstetricians in both public and private sector hospitals have demonstrated confidence in using the Raabta program to support pregnant women at HRPE. They mentioned that the Raabta program dashboard is easy to use and provides an opportunity to view all high-risk patient data on one platform. However, telehealth experts, and obstetricians in the public sector identified various factors that might impact their willingness to use the program, such as increased patient volumes, inadequate logistics support including the shortage of trained staff, and a lack of incentives.

Someone like me will be very excited, …But a clinician? Especially working in a government hospital. This is something which is going to bother him. This is a burden on him. This is a liability on him. Unless he or she is being compensated. (Telehealth Expert 04)

### Domain 2: intervention characteristics

Obstetricians acknowledged that the evidence-based guidelines (NICE) forming the basis of the Raabta program are well-known and accepted. Most interviewees perceived the remote-monitoring aspect of the Raabta program as convenient, empowering, systematic, and clinically effective compared to the current standard of care, where women face difficulty in booking appointments outside of their regular scheduled visits, and the quality of blood pressure measurement in the clinic is often deemed inappropriate.

See, the problem in pregnancy is that every 6 hours, … you cannot run to the doctor. Sometimes, after two weeks, you get an appointment, so all that time, if … no one is guiding you…then it will cause problems to baby's health like pre-term delivery. To avoid all those things, I think it's important that you get a notification so that you know the next step …the notification will work like an alarm for women. (Private Sector Patient 07)

Participants provided feedback on how they perceived the Raabta program and suggested changes. The layout of the Raabta system was described by healthcare professionals as having a user-friendly, intuitive, and neat design. Participants appreciated the context-specific design features of the Raabta program for patients with low digital literacy and language barriers, such as voice instructions, visual cues, and color-coded alerts.

Very good, even if illiterate people are also there, that is, those who are not so educated can use it through voice notes. Some people are not able to read, so they can communicate through the voice note. This is a very good way to facilitate communication; it allows them to use the technology and share their opinions. (Private Sector Patient 04)

A potential barrier was that the patient-facing Raabta app does not cater to other commonly spoken languages such as Sindhi and Pashto. Patients, obstetricians, and telehealth experts recommended adapting the patient-facing Raabta app for language inclusivity to ensure the program’s widespread adoption among diverse populations.

Yes, the language that is used in the application is Urdu. So if you use it in Pakistan, then Pakistan is a very diversified country with respect to language. So if we can add any other language other than Urdu, it would be very good. For example, if someone is living in interior Sindh and cannot understand the Urdu instructions, they might know Sindhi, so this would be beneficial. (Public Sector Patient 06)

Additionally, obstetricians from both the public and private sectors mentioned that the Raabta program lacks a complete set of diagnostic parameters for PE, such as symptoms of nausea/vomiting, swelling in limbs, and tests for proteinuria. Providers expressed concern that the current parameters, especially those related to PE symptoms, could lead to false red alerts, resulting in unnecessary hospital visits and complexity in diagnosing the PE condition. For example, pregnant women often complain of headaches, which could be unrelated to the PE condition and might trigger unnecessary red alerts.

I want to emphasize that a headache is a very common symptom. For instance, a woman who already has 2 or 3 kids, and may work as a domestic worker or in a field, will come home tired. She will experience headaches due to various factors… Consequently, she will keep receiving red alerts despite being in a normal condition. In such cases, both the doctor and the patient will bear an additional burden due to unnecessary hospital visits. (Decision Maker 07)

All groups of respondents highlighted the need for a pilot trial to assess the feasibility of implementing the Raabta program in both public and private sector hospitals. All key informants emphasized the need to generate initial data/evidence regarding the program’s efficacy through the pilot trial.

I suggest conducting a pilot test and performing this testing with patients having various literacy backgrounds, technological literacy, cultural norms, and family dynamics as well as across different age groups. (Telehealth Expert 05)

Most participants perceived the Raabta program as easy to use and navigate. However, a few public sector patients, telehealth experts, and obstetricians highlighted challenges with using the Raabta app, including limited digital literacy, poor internet connectivity in rural areas, and the lack of access to personal mobile phones for some pregnant individuals.

I think it can be difficult because, being an online application, it's not easily understood, especially by uneducated people who don't know how to read or submit readings. In some rural areas, there are internet issues, unlike urban areas, where network facilities are better. Also, not everyone has a phone—sometimes only one person in the household has a phone, and if the pregnant person doesn’t have it, tracking and sharing data with the doctor becomes a challenge. (Public Sector Patient 03)

Some telehealth experts highlighted some technical challenges associated with the Raabta program usage, including issues with placing the blood pressure cuff correctly on the arm, Bluetooth connectivity, and battery replacement in the blood pressure device. These issues were identified as potential deterrents affecting women's motivation to engage with the program. Additionally, obstetricians in both public and private sectors expressed concerns about patient safety in relation to incomplete daily tasks and unresponsiveness to alerts, which might impact the overall effectiveness of the program in ensuring timely intervention and patient well-being.

If she is not reaching the hospital, even after the alert has been sent to her, then it's concerning. I don't know how we could reach out to that woman—should we call her? Or should we call her family or send someone? How would we go about it? It is a threat to patient safety. (Public Sector Obstetrician 01)

Telehealth experts and obstetricians also highlighted the additional financial burdens associated with the use of the Raabta program. Obstetricians explained that continuous monitoring could cause hospital admissions, blood tests, medication prescriptions, and transportation costs. These financial considerations could potentially impede the widespread adoption of Raabta.

only after you study would you be able to know, the extra cost, let's say, once you see a patient with high-risk factors. And they get a yellow alert, and then after 4 hours, they again have raised blood pressure. They come to the hospital. They get investigated for all the tests for PE. … So it's not the cost of the BP app or the cost of the mobile phone because that is a one-time expenditure. Right, but … the cost for all those investigations.(Private Sector Obstetrician 03)

### Domain 3: inner setting

The two sectors included in this study are organized and function differently. The public sector encounters challenges related to physical space and limited human and financial resources, posing constraints on the implementation of the Raabta program at public sector hospitals. In contrast, the private sector is well-equipped and can leverage existing infrastructure and resources, such as physical space, logistical support, and nursing staff, for the implementation of the Raabta program. For instance, nurses in the private sector can be involved in monitoring alerts through the Raabta program dashboard.

At XYZ public hospital, we are understaffed. … Therefore, installing a remote monitoring system, having nurses consistently oversee that platform, and reaching out to women based on specific alerts, would be a challenge. (Public Sector Obstetrician 01)

A majority of obstetricians in both the public and private sectors share the perception that the current state demands a shift, particularly in efficiently screening high-risk pregnant women for PE. These obstetricians acknowledged the Raabta intervention for its capacity to enable remote monitoring and recording of high blood pressure and PE, offering an improved approach. However, resistance to change has surfaced among obstetricians in the public sector regarding the implementation of the Raabta program. This reluctance is rooted in the belief that adopting the program could add to their high workload and place additional strain on their resources. Decision-makers and telehealth experts noted that there may be hesitancy among the public sector to implement the Raabta program due to resource and funding constraints.

… Not every piece of data has to be monitored by a physician, and not every call has to be answered by a physician. A multiple-tiered system can be established where different kinds of health providers are involved, making it cost-effective. I think this is definitely something that the government can and should support. (Telehealth Expert 02)

Obstetricians, decision-makers, and telehealth experts highlighted some factors to strengthen the implementation climate for Raabta. Telehealth experts, obstetricians, and decision-makers stressed the importance of having support and endorsement for the Raabta program from department leads to gauge interest and willingness from obstetricians in both the public and private sectors. Obstetricians in both the public and private sectors raised concerns regarding monitoring the clinician dashboard. They felt that they were too busy with their clinical duties and other academic work, making it challenging to continuously monitor alerts on the Raabta dashboard. The obstetricians suggested having a dedicated resource for monitoring the clinician dashboard and responding to alerts. In cases where obstetricians are assigned monitoring duties, it was suggested that obstetricians need to be incentivized for their active participation in the Raabta program.

But in terms of clinicians, things are quite different because it always depends on their workload. Most OBGYNs have a lot of patients, and they don’t have time for anything like this. The point is, what's in it for me? This is a question everyone will ask when talking to clinicians. So, if it is tied to their revenue, then it could definitely be helpful. (Telehealth Expert 05)

### Domain 4: outer setting

Obstetricians highlighted current practices for screening high-risk pregnant patients for PE risk. In the private sector, patients consistently seek care and are less prone to complications, including those associated with PE. However, in the public sector, patients encounter challenges in adhering to regular antenatal care visits due to sociocultural and financial constraints, leading to insufficient screening for PE and complications, including death.

Just over the last hour, I was talking to my research officer dealing with a woman with high blood pressure at a local public health facility. She was told that she needed to go to JPMC immediately because of her blood pressure, but she decided to go home first, and she died on the way. We just lost her, even though we provided her with transport to go to JPMC, but she insisted on going home first. (Telehealth Expert 06)

Obstetricians in both sectors often request high-risk patients to record blood pressure and PE symptoms. While some patients demonstrate high motivation and responsibility in adhering to these instructions, particularly in the private sector, others, especially in the public sector, do not comply. As a result, obstetricians perceive that patients in the public sector are likely to lack compliance with the Raabta program. The consensus among obstetricians and telehealth experts is that the Raabta program would significantly address the needs of high-risk pregnant women and reduce their anxiety during the early stages of pregnancy. However, a potential challenge lies in ensuring pregnant patients’ compliance with recording their blood pressure and symptoms every day on the Raabta app.

One thing that I can tell you from experience is, the compliance of the patient to this will be a challenge. … One thing that you can add is a notification or a pop-up or a ringer if by a certain time of the day they haven't recorded their blood pressure because people forget, women forget. (Telehealth Expert 03)

Decision-makers highlighted that internationally and nationally, maternal and child health initiatives receive priority over other programs in terms of funding and general support, suggesting a favorable external policy environment for the spread and scale of Raabta.

Maternal and child health are always a priority for funding. I mean, those are high-priority health issues, and I don't think that you will need much effort to convince. This is already high on their priority list, and there is already a supportive policy landscape around this. (Telehealth Expert 02)

### Domain 5: implementation process

Participants, including obstetricians, decision-makers, and telehealth experts, were asked to elaborate on their thoughts regarding how to implement and sustain the Raabta program in public and private sector hospitals in Pakistan. Obstetricians proposed the adoption of a nurse-led model to implement the Raabta program. Their recommendation involved engaging nurses, and paramedics to train pregnant women in the use of the program and monitor alerts on the Raabta dashboard. According to obstetricians, the nurse-led model would mitigate challenges related to continuous alert monitoring, offering a practical solution to enhance the program's effectiveness. In addition, decision-makers suggested that considerable efforts should be made during pilot implementation to consider how the Raabta intervention can be sustained within existing maternal and child health service packages. Specifically, telehealth experts suggested considering the integration of Raabta into existing digital platforms launched by the Ministry of Health. Finally, obstetricians and telehealth experts recommended prescribing Raabta during antenatal visits as a strategy to enhance the uptake and use of Raabta among high-risk pregnant women.

…Every large hospital is affiliated with a medical college, and they have students. You can utilize this resource by identifying a person from the current batch who will be very happy and excited to be part of the team. Alongside this, consider involving student nurses, registered nurses, dressers, or paramedics, depending on the hospital's structure. (Private Sector Obstetrician 05)

The participants also highlighted the need to engage key opinion leaders, such as clinical champions, medical institutions, and decision-makers, for the success of the Raabta pilot program and future large-scale trials. In addition, telehealth experts suggested establishing an advisory board/committee comprising clinical champions, policymakers, celebrities, telehealth experts, hospital department heads, and leaders from international organizations to garner support for the sustainability of the Raabta program.

You need to have the right people interested … key champion doctors who can talk about it. The conversation starts at dinner, it starts on calls, it starts with lunches. Eventually, when you have some data to show, eventually when you have a league of doctors interested in championing you, and eventually when the patients see that they're being helped, then you have enough material to influence policy … So, I think the first thing you need to do is get a great number of doctors endorsing it. (Telehealth Expert 03)

## Discussion

### Principal findings

The study used the CFIR to identify key aspects of potential Raabta implementation including facilitators and barriers, both in public and private sector hospitals in Pakistan. Overall, the study participants perceived the Raabta program as user-friendly and valuable for supporting pregnant women at HRPE and their caregivers in both public and private healthcare settings. The positive attitude towards Raabta, observed among both public and private sector patients, obstetricians, telehealth experts, and decision-makers, highlights its potential to improve current practices and patient outcomes. Despite public sector patients’ readiness to utilize the Raabta program for early pregnancy complication identification, prompt treatment, care continuity, and self-management, they face sociocultural and financial barriers in engaging with digital health technology, such as limited smartphone access, digital and health literacy, and poor language proficiency. Additionally, obstetricians in both sectors voiced concerns that incomplete daily tasks and unresponsiveness to alerts could compromise patient safety and the program's effectiveness. A majority of obstetricians in both the public and private sectors share the concern that the current state demands a shift, particularly in efficiently screening high-risk pregnant women for PE. The implementation process-related factors identified in this study, particularly regarding feasibility assessment, align closely with findings from other TM studies.^40–44^ The multifaceted insights gained from the study provide critical considerations for optimizing intervention characteristics, outer setting, inner setting, individual characteristics, and the implementation process for Raabta program implementation across diverse healthcare settings.

### Potential facilitators for implementing raabta

All participants were generally positive about the Raabta program and acknowledged that the evidence-based recommendations (NICE guidelines)^32^ forming the basis of the technology are well-known and accepted. They found the technology to be easily navigable even for public sector patient participants with low digital and language literacy, attributing it to context-specific design elements such as voice instructions and color-coded alerts. This contrasts with the common assumption in most TM programs, where operational designs presume a basic level of digital literacy among patients. Such assumptions restrict the utilization of valuable digital tools for individuals with low health and digital literacy.^40,45–47^ In our commitment to foster inclusivity, we addressed this limitation by developing the culturally sensitive Raabta program in consultation with expert obstetricians and researchers in Pakistan who possess a deep understanding of the local context and the unique needs of high-risk pregnant women.

Another implementation facilitator identified in our study is the presence of a favorable external policy and funding landscape for Raabta. This is particularly beneficial as the program falls under the broader framework of maternal and child health, typically supported through global health initiatives and developmental agendas. This finding is consistent with Creanga et al.'s study which conducted a secondary analysis of cross-sectional data from 113 LMICs to assess the maternal health policy environment's impact on service utilization. The study observed a modest association between countries with a more favorable policy environment for maternal health and increased utilization of antenatal care, institutional delivery, and postnatal care services among mothers in LMICs.^48^

Obstetricians expressed confidence in using the Raabta dashboard for monitoring alerts and viewed the Raabta program as potentially advantageous for both patients and providers, with the potential to alleviate the burden on healthcare resources, including reducing emergency room visits in the public sector healthcare system and shortening hospital stays for patients compared to the current standard of care. This is in line with Fazal et al.'s study, which focused on designing and evaluating text-based remote monitoring technology (Florence) for monitoring pregnant patients with pregnancy-induced hypertension in the National Health Service (NHS) England. As per NICE guidelines, the NHS monitors patients with mild hypertension once weekly and patients with moderate hypertension twice weekly. This leads to more than 10,000 attendances per year to the maternity day assessment unit, of which 22% were purely for BP monitoring, causing a burden on NHS maternity services. The study confirmed that the Florence technology alleviated the burden on NHS providers, thereby making them available for managing more complicated pregnancy cases.^49^

### Potential barriers for implementing raabta

Despite the participants’ positive attitude towards the Raabta program, several potential barriers to its implementation were identified, including potential technical challenges associated with Raabta program use and the lack of inclusion of other commonly spoken languages such as Sindhi and Pushto, highlighting the need for language adaptability to ensure widespread adoption among diverse populations seeking care at public sector hospitals. Ko SQ et al.'s study demonstrated how language and cultural barriers can be overcome through collaboration and iteration in designing a remote mHealth solution for reporting vital parameters during COVID-19. The researchers collaborated with culturally and linguistically diverse, low-income dormitory residents in Singapore and iterated a simple, interactive chatbot app accessible in multiple languages, including Bengali, Tamil, Mandarin, or Hindi to facilitate rapid adoption of the app.^50^

A significant barrier is the disparity in access to essential technologies between public and private sector patients. Private sector patients generally have better access to smartphones, internet services, and home blood pressure (BP) monitors, which may facilitate smoother future engagement with the Raabta program. In contrast, public sector patients often lack these resources, potentially limiting the program's feasibility and effectiveness in public hospitals. The absence of smartphones, internet access, and home BP monitors can hinder communication and adherence. Addressing this gap may require future initiatives to provide additional resources, subsidies, or alternative solutions to ensure equitable program adoption across both sectors.

The prospect of additional costs was recognized as a potential barrier, as continuous monitoring might incur extra expenses, potentially limiting the adoption and utilization of the program. The economic evaluation of a similar intervention, CLIP, in Pakistan, which enabled CHWs to use the PIERS On the Move mHealth app for risk stratification, reported that it was not cost-effective in reducing adverse maternal and perinatal outcomes.^51^ However, Lanssens et al.'s scoping review on the effectiveness of TM in obstetrics identified two studies^52,53^ in high-income settings, demonstrating that remote prenatal monitoring for women with gestational hypertensive diseases can significantly reduce healthcare costs compared to conventional care.^7^ Future studies should consider indirect costs such as transportation and travel, along with other expenses associated with frequent monitoring, such as diagnostic tests, to comprehensively capture considerations for patient costs.

Obstetricians across both public and private health sectors reported that monitoring through the dashboard would be challenging due to their existing clinical workload and suggested the need to have a dedicated resource for monitoring the clinician dashboard and responding to alerts. A similar finding was found in a study by Medich et al., which explored the use of passive mobile monitoring and self-tracking among patients with serious mental illness in Los Angeles. The study identified obstetricians’ concerns regarding increased workload and alert fatigue tied to dashboard data review. Similar to our study, providers in this study suggested the need for more physicians who can share the patient load.^54^ Future research should explore alternative approaches, such as employing technicians or paramedics as mediators between obstetricians and patients for monitoring alerts to ease the adoption of interventions and alleviate potential burdens for providers associated with introducing new interventions.^55^

### Key differences in barriers and facilitators in public and private health sector

When evaluating the potential implementation of the Raabta program across public and private sector hospitals, several factors emerged that could influence how the program is adopted. Differences in patient demographics, health and digital literacy, cultural norms, obstetrician readiness, hospital infrastructure, and resource constraints are likely to affect the success of implementation in each sector. Similar to our study, Shaikh et al. studies highlighted significant disparities between patients in public and private health sectors regarding access to resources, cultural norms, health-seeking behaviors, and socioeconomic status, all of which significantly impact healthcare service utilization among public sector patients in Pakistan.^56,57^ In the context of the Raabta program, public sector patients may face greater barriers in engaging with the program due to limited resources and support. Meanwhile, private sector patients are more likely to adopt the Raabta program, benefiting from better health and digital literacy, financial support, and positive health-seeking behaviors. Consistent with our findings, Ahmed et al.'s study^58^ identified the inherent demographic, socioeconomic, cultural, financial, and accessibility barriers faced by public sector patients in seeking care and utilizing digital health technologies.

In line with our study's findings regarding the influence of limited health literacy, poor language proficiency, and cultural norms on patient engagement with the Raabta program, Kemp et al.'s study on digital health technology implementation in cancer care in Australia found that limited digital and traditional health literacy were identified as major barriers to digital technology engagement, with a range of difficulties identified for older, younger and socio-economically or geographically disadvantaged.^59^ Evidence suggests that investments are required to address digital and health literacy needs across the spectrum of engagement including education and training to enhance user ability and designing and tailoring digital health approaches to suit individual needs, particularly for those living with socioeconomically disadvantaged circumstances and those with lower levels of digital literacy.^59,60^

In terms of obstetricians’ willingness to use the Raabta program, our study revealed resistance to change among public sector obstetricians due to the belief that the Raabta program would add to their workload and place additional strain on their resources. Similar to our study, Hashmi et al.'s study on using mHealth to improve diabetic guidelines adherence concluded that physicians in public teaching hospitals of Pakistan were not consistent with the use of mHealth intervention due to workload, time constraints, and absence of incentives.^61^ These factors emphasize the importance of carefully designing the implementation to reduce the burden on healthcare providers, particularly in public settings, through strategies like incentive models or integration with existing workflows.

Regarding hospital infrastructure and resources, while private sector hospitals are generally well-equipped and capable of leveraging existing infrastructure and resources for implementing digital technologies, the public sector faces challenges due to insufficient physical space and limited human resources. This is supported by Kazi et al.'s mixed-method study which analyzed digital health projects conducted in Pakistan over the last 5 years.^62^ The study found equal representation of such projects from both the public (17/51, 34%) and private (17/51, 34%) sectors. However, the strengths, weaknesses, opportunities, and threats (SWOT) analysis conducted in the study highlighted a lack of capacity in public health facilities such as basic health units, rural health units, and tertiary hospitals to incorporate digital health interventions, resulting in fragmented and vertical efforts.^62^ A strategic approach, such as pilot testing and phased rollouts, could help overcome these infrastructure barriers. Despite these differences, there was consensus among public and private sector patients, obstetricians and telehealth experts that the Raabta program holds significant promise in addressing the unique needs of high-risk pregnant women.

### Future directions for implementation

There were specific recommendations from this study to guide the future implementation of the Raabta program. First, obstetricians and telehealth experts recommended launching a pilot trial in both the public and private sectors to evaluate the feasibility of the Raabta program. To ensure the success of this pilot trial, it was recommended to secure support and endorsement for the Raabta program implementation from department leaders, gauge interest and willingness among obstetricians in both sectors and establish an advisory committee involving key opinion leaders. Second, to ensure the Raabta program sustainability, obstetricians in the private sector recommended adopting a nurse-led model and leveraging the expertise of nurses and paramedics for training pregnant women and monitoring the Raabta dashboard. Decision-makers in the public sector proposed exploring integration with existing digital platforms established by the Ministry of Health for long-term sustainability. Lastly, to increase the feasibility of implementation, it was recommended that obstetricians in both sectors should prescribe Raabta during routine prenatal care visits to enhance the uptake of the Raabta program among pregnant patients.

### Strengths and limitations

The study possesses several strengths and limitations. First, the strength lies in the utilization of the CFIR to enable the understanding of barriers and facilitators associated with Raabta implementation across the public and private sectors in Pakistan. Second, the study interviewed a diverse group of participants from both the public and private sectors, including patients, obstetricians, decision-makers, and telehealth experts, to gain a comprehensive understanding of potential barriers and facilitators influencing Raabta implementation in Pakistan. Finally, the study offers practical insights for future implementation efforts in the public and private sectors, such as adopting a nurse-led model and exploring the integration of Raabta with existing digital platforms. However, this study is also subject to certain limitations. The study recruited obstetricians and patients from a public and a private hospital in Pakistan. However, it is important to note that all private sector patients had personal access to smartphones and the internet, which may not be the case for all private sector patients in other hospitals. Thus, the findings of the research may not fully represent all public and private sector patients and obstetricians, especially those in different cities with distinct socio-cultural backgrounds, financial means, and healthcare infrastructure and resources. Finally, despite employing purposive and snowball sampling to recruit telehealth experts and decision-makers, the study may have missed perspectives from certain stakeholder groups including nurses, insurers, and regulators, within the public and private sectors.

## Conclusion

This study explored evidence of the barriers and facilitators associated with implementing the Raabta program in public and private sector hospitals in Pakistan, guided by the CFIR framework. The study revealed that both public and private sectors are organized and function differently, suggesting that Raabta implementation may be more feasible in the private sector due to patient demographics including language proficiency, advanced health and digital literacy, supportive cultural norms, financial resources, physician readiness to use the Raabta program, and availability of hospital infrastructure and resources. The study highlights the contextual nuances that will shape the landscape of Raabta program implementation in both public and private sectors in Pakistan. The identification of potential challenges and opportunities will be instrumental in developing future customized strategies for Raabta program implementation in both public and private sector hospitals.

## Appendix. Semi-structured interview guide.

**Table:**

Interview guide questions as per CFIR domains
*Domain I: Intervention Characteristics*
1. What do you (clinicians and other stakeholders) think of the Raabta program? [Evidence Strength & Quality]
2. What kind of evidence or proof is required to convince staff about the effectiveness of the Raabta program? [Evidence Strength & Quality]
3. How does the Raabta program compare to other similar existing programs? [Relative Advantage]
4. What kinds of changes or alterations are needed to make the Raabta program work effectively at public and private sector hospitals in Pakistan? [Adaptability]
5. How difficult is the Raabta program, including the Raabta app and clinician dashboard? [Complexity]
6. What do you think about the way the Raabta program is designed and presented? [Design Quality & Packaging]
7. What supports are available to help you implement/ use the Raabta program? [Design Quality & Packaging]
8. What cost will be incurred to implement the Raabta program? [Cost]
9. What are your thoughts about the Raabta program first being tested on a small scale? [Trialability]
10. What are your thoughts on the safety and privacy issues associated with the use of the Raabta program?
*Domain II: Inner Setting*
11. How does the current infrastructure of your organization (social architecture, age, maturity, size, or physical layout) influence the implementation of the Raabta program? [Structural Characteristics]
12. What do you think would be the general receptivity in your organization to implementing the Raabta program? [Implementation Climate]
13. What is the underlying need that drives the necessity of implementing the Raabta program[Tension for Change]
14. How likely is it that the implementation of the Raabta program will be less important compared to other important initiatives happening in your organization? [Relative Priority]
15. What kinds of organizational incentives are there to help ensure that the implementation of the Raabta program is successful? [Organizational Incentives & Rewards]
16. How well does the Raabta program fit with existing work processes and practices in your setting? [Compatibility]
*Domain III: Outer Setting*
17. How well do you think the Raabta program will meet the needs of high-risk pregnant women? [Patient Needs & Resources]
18. What barriers will the pregnant women face to participating in the Raabta program? [Patient Needs & Resources]
19. Can you tell me what you know about any other organizations that have implemented similar programs? [Peer Pressure]
20. What kind of local, state, or national performance measures, policies, regulations, or guidelines will influence the decision to implement the Raabta program? [External Policies & Incentives]
21. What kind of financial or other incentives are available to implement/deliver/sustain the Raabta program? [External Policies & Incentives]
*Domain IV: Characteristics of Individual*
22. Do you think the Raabta program will be effective in your setting? 23. Why or why not? [Knowledge & Beliefs about the Intervention]
24. How do you feel about the Raabta program being used in your setting? 25. Do you have any feelings of anticipation? Stress? Enthusiasm [Knowledge & Beliefs about the Intervention]
26. How confident are you that you will be able to successfully implement/use the Raabta program? [Self-efficacy]
27. How would you describe your personal readiness for the Raabta program and its implementation/use? [Individual Stage of Change]
*Domain V: Process*
28. Who are the key influential individuals to get on board with this implementation? [Opinion Leaders]
29. Other than the formal implementation leader, are there people in your organization who would likely to champion the Raabta program?[Champions]
30. How would you assess progress towards implementation of Raabta program? 31. [Reflecting & Evaluating]
32. What kind of evaluation information would you plan to collect if you were to implement the Raabta program? [Reflecting & Evaluating]
